# A Focus on Reward Prediction and the Lateral Habenula: Functional Alterations and the Behavioral Outcomes Induced by Drugs of Abuse

**DOI:** 10.3389/fnsyn.2018.00012

**Published:** 2018-05-29

**Authors:** Nicholas M. Graziane, Peter A. Neumann, Yan Dong

**Affiliations:** ^1^Departments of Anesthesiology and Perioperative Medicine and Pharmacology, Penn State College of Medicine, Hershey, PA, United States; ^2^Department of Psychiatry and Behavioral Sciences, Stanford University School of Medicine, Stanford, CA, United States; ^3^Departments of Neuroscience and Psychiatry, University of Pittsburgh, Pittsburgh, PA, United States

**Keywords:** lateral habenula, addiction, reward, learning, drugs of abuse, neurocircuits

## Abstract

The lateral habenula (LHb) regulates reward learning and controls the updating of reward-related information. Drugs of abuse have the capacity to hijack the cellular and neurocircuit mechanisms mediating reward learning, forming non-adaptable, compulsive behaviors geared toward obtaining illicit substances. Here, we discuss current findings demonstrating how drugs of abuse alter intrinsic and synaptic LHb neuronal function. Additionally, we discuss evidence for how drug-induced LHb alterations may affect the ability to predict reward, potentially facilitating an addiction-like state. Altogether, we combine *ex vivo* and *in vivo* results for an overview of how drugs of abuse alter LHb function and how these functional alterations affect the ability to learn and update behavioral responses to hedonic external stimuli.

## Introduction

**Figure 1:**
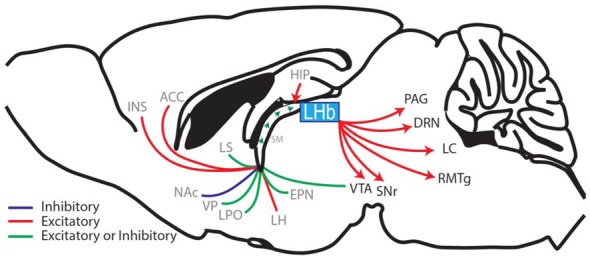
Sagittal rodent brain section illustrating the afferents and efferents of the lateral habenula (LHb). The LHb interconnects the forebrain with the midbrain and hindbrain regions by receiving afferents through the stria medullaris (SM), which contains fibers from the hippocampus (HIP), ventral pallidum (VP), lateral hypothalamus (LH), entopeduncular nucleus (EPN; the EPN is equivalent to the globus pallidus interna in primates and humans and sends a prominent afferent to the LHb (Hong and Hikosaka, 2008; Wallace et al., 2017)) diagonal band of Broca (cholinergic nerve fibers, not shown), lateral septum (LS), lateral preoptic area (LPO), ventral tegmental area (VTA; Stamatakis et al., 2013; Lammel et al., 2015), nucleus accumbens (NAc), anterior cingulate cortex (ACC) and the anterior insula (INS; afferent brain regions labeled in gray). The LHb then relays information via glutamatergic efferents that together form the fasciculus retroflexus. These efferents project to the substantia nigra (SNr), VTA, rostral medial tegmental nucleus (RMTg), the dorsal raphe nuclei (DRN), the locus coeruleus (LC) and the periaqueductal gray (PAG) (LHb-projecting brain regions labeled in black; Aizawa et al., 2011; Shelton et al., 2012; Quina et al., 2015; Yetnikoff et al., 2015).

The ability to accurately predict rewarding or aversive outcomes throughout life is a critical adaptive behavior that promotes survival and allows avoidance of threatening or unpleasant confrontations. This adaptive ability is attributed to an evolutionarily conserved hedonic neurocircuit which consists of an interwoven network linking forebrain, midbrain and hindbrain regions, that together systematically regulate the release of monoamines (neuromodulators that mediate reward prediction and motivated behaviors; Kelley, 2005). The lateral habenula (LHb) is centrally located within this hedonic neurocircuit (Figure 1; Aizawa et al., 2011; Shelton et al., 2012) and regulates monoamine release and monoamine controlled behaviors including pain modulation, sleep, stress, motor behaviors and the focus of this review, reward learning (Lecourtier and Kelly, 2007; Hikosaka, 2010; Evely et al., 2016; Boulos et al., 2017).

Reward learning crucially relies on midbrain dopamine neuron activity. This neuronal activity is precisely timed with reward predictions (expected reward) and reward outcomes (Schultz et al., 1997; Steinberg et al., 2013; Chang et al., 2017; Schultz, 2017). If the reward received is better or greater than the predicted reward, it is referred to as a positive reward prediction error (RPE) and is associated with increases in dopamine neuron firing (Schultz et al., 1997). On the other hand, if the reward received is worse or less than the predicted reward, it is referred to as a negative RPE and is associated with decreases in dopamine neuron firing (Schultz et al., 1997). The LHb is linked to this process, as stimulating neurons in the LHb strongly inhibits midbrain dopamine neurons (Christoph et al., 1986; Ji and Shepard, 2007; Matsumoto and Hikosaka, 2007). Additionally, the LHb acts as a center for negative RPEs, as subpopulations of neurons respond to negative prediction errors with neuronal excitation, and to positive prediction errors with neuronal inhibition (Bromberg-Martin and Hikosaka, 2011).

This review discusses current evidence that links the LHb to reward prediction by focusing on the LHb’s activation patterns and regulation of efferent targets during exposure to hedonic stimuli. Additionally, we discuss the underlying mechanisms related to drug-induced changes in LHb functional output, and discuss evidence for how drug-induced LHb alterations may affect the ability to predict reward, potentially facilitating an addiction-like state.

## How Does the LHb Regulate Reward Prediction and Reward Learning?

The LHb regulates reward prediction and reward learning, in part, through its glutamate projections to the rostromedial tegmental nucleus (RMTg), a brain region that sends inhibitory input to dopamine neurons in the ventral tegmental area (VTA). During negative RPEs, LHb neurons are activated and send excitatory signals to RMTg inhibitory neurons which project to and inhibit dopamine neuron firing (Matsumoto and Hikosaka, 2007; Jhou et al., 2009; Kaufling et al., 2009; Lammel et al., 2012; Figure 1). The LHb’s control over midbrain dopamine neurons is significant because inhibition of dopamine neuron firing is causally associated with negative RPEs and extinction learning (Steinberg et al., 2013; Chang et al., 2016, 2017). Furthermore, LHb activity does not correlate only with negative valence, but also acts as a center for negative RPE (Ullsperger and von Cramon, 2003; Salas et al., 2010; Bromberg-Martin and Hikosaka, 2011), as LHb neurons respond to reward information that is unexpectedly cued, delivered, or denied (Bromberg-Martin and Hikosaka, 2011). Thus, it appears that the LHb receives and processes negative RPE information in much the same way that dopamine neurons process positive RPE signals.

Based on the LHb’s role in processing negative RPE signals, it follows that LHb activity is critically involved in learning and memory processes that are associated with avoidance behaviors (Stamatakis and Stuber, 2012). As discussed earlier, the LHb regulates reward prediction through its glutamate projections to the RMTg. In addition, the LHb sends direct glutamate projections to the VTA, which, when activated, promote avoidance behaviors (Lammel et al., 2012). Light stimulation of LHb neurons that project directly to the VTA causes strong conditioned place aversion, with evidence suggesting that VTA neurons receiving direct excitatory connections from the LHb preferentially project to the medial prefrontal cortex (Lammel et al., 2012). Since a subpopulation of VTA dopamine neurons are activated during exposure to noxious stimuli (Brischoux et al., 2009; Bromberg-Martin et al., 2010), these results suggest that the LHb may promote aversive behaviors through neurocircuit pathways that include specific subpopulations of dopamine neurons.

Examining loss-of-function in the LHb shows that inhibiting LHb function with habenular lesions in rats impairs their ability to recognize or learn newly introduced escape routes in a forced swim test (Thornton et al., 1985), and also impairs their ability to learn to avoid a foot shock during an active avoidance task (Thornton and Bradbury, 1989). Bilateral lesions in rats impair inhibitory avoidance acquisition (latency to leave an enclosed elevated T-maze arm and enter a non-enclosed arm), though panic and escape responses appear to be normal (Pobbe and Zangrossi, 2008). Also, habenula lesions in the rat prevent learned helplessness in a classical foot shock paradigm (Amat et al., 2001). By reducing LHb output to the dorsal raphe, the classically observed increase in serotonin signaling during learned helplessness is attenuated, preventing the formation or expression of learned helplessness behavior (Amat et al., 2001). Lastly, LHb lesions in mice cause dopamine RPE signals to no longer respond specifically to reward omissions, and also cause dopamine RPEs to subsequently lose the ability to signal graded levels of reward reliably, though a significant response still remains (Tian and Uchida, 2015). Together, studies examining loss-of-function in the LHb show evidence of impaired learning, particularly to aversive stimuli. However, work continues to dissect the distinct contribution of the LHb to conditioned-learning processes, as the LHb may participate in aversive learning but not punishment-associated avoidance behaviors (Jean-Richard Dit Bressel and McNally, 2014; Zapata et al., 2017).

## Does Localized Drug Administration Alter LHb Neuronal Function?

Subpopulations of neurons in the LHb are differentially regulated by cocaine, a dopamine uptake blocker and one of the commonly abused drugs. LHb neurons that increase their firing during a nociceptive tail pinch or neurons that are non-responsive (non-nociceptive) to a tail pinch have decreases in firing rates during cocaine microiontophoresis application (Dougherty et al., 1990). In contrast, LHb neurons that decrease their firing during a nociceptive tail pinch have increases in firing rates during localized cocaine administration (Dougherty et al., 1990). Cocaine-induced increases in LHb neuronal firing rates are likely due to dopamine-dependent increases in inward currents, dopamine-dependent increases in glutamatergic synaptic transmission, and decreases in GABA presynaptic release (Good et al., 2013; Zuo et al., 2013). In addition to decreasing GABA presynaptic release, localized cocaine administration increases GABA presynaptic release on subpopulations of LHb neurons (Zuo et al., 2013), which may contribute to the above mentioned cocaine-induced decreases in LHb neuronal firing.

Localized ethanol administration, similar to cocaine, increases LHb neuronal firing and enhances presynaptic glutamate release in a process that is regulated through dopamine D1-receptor activation (Zuo et al., 2017a). However, this increase in LHb neuronal firing is limited by ongoing GABA_A_R-mediated inhibition, which is also enhanced by ethanol and is likely caused by dopamine-dependent pre and postsynaptic modifications (Zuo et al., 2017b). Furthermore, the effects of ethanol on spontaneous excitatory and inhibitory postsynaptic currents (sEPSC and sIPSC, respectively) vary among LHb neurons. Some neurons undergo differential potentiation of sEPSCs and sIPSCs, other neurons show changes in sEPSCs and sIPSCs that are inversely related, and yet different sets of neurons do not undergo any changes in sEPSCs or in sIPSCs (Zuo et al., 2017b).

Finally, localized nicotine administration depolarizes LHb neurons and increases their firing activity (Zuo et al., 2016). At the synapse, nicotine enhances presynaptic glutamate release and enhances GABAergic transmission through pre and postsynaptic modifications (Zuo et al., 2016).

Taken together, these results demonstrate that localized application of addictive substances in the LHb influences a wide array of neuronal properties including firing rates and excitatory and inhibitory transmission, often with diverse effects among subpopulations of neurons. Although these subpopulations have yet to be fully characterized, one potential distinguishing characteristic is the LHb’s projection targets, as data suggest that LHb neurons projecting to specific brain regions have selective responses to drugs of abuse (Maroteaux and Mameli, 2012; Good et al., 2013).

## Does Drug Withdrawal Alter LHb Neuronal Function?

LHb neuronal firing rates (measured *in vivo*) are decreased shortly after cocaine administration (0–10 min post injection), but this transient inhibition progresses to increased firing activity (15–35 min post injection) in specific neuronal populations (~30% of the recorded neurons; Jhou et al., 2013), which parallels cocaine’s immediately rewarding, but later aversive, sensations (Ettenberg et al., 1999). In *ex vivo* electrophysiological measurements, a subpopulation of LHb neurons shows decreases in excitability during cocaine bath application, which then progresses to increases in excitability above the original baseline after cocaine is washed out (Jhou et al., 2013). These results are in agreement with the observed *in vivo* biphasic LHb neuronal firing patterns that occur in a subset of neurons during intravenous cocaine administration (Dougherty et al., 1990; Jhou et al., 2013).

In addition to the effects on LHb neurons following acute cocaine exposure, withdrawal from short-access (2 h/day) cocaine self-administration causes increases in LHb neuron excitability as measured using *ex vivo* electrophysiology (Neumann et al., 2014). In animals after 5–7 days of withdrawal from cocaine self-administration, regular spiking LHb neurons have a significantly higher membrane excitability compared to saline-exposed controls (Neumann et al., 2014), and the increased membrane excitability returns to basal levels by withdrawal day 45 (Neumann et al., 2014).

In addition to changes in LHb neuronal firing induced by withdrawal from drugs of abuse, synaptic changes at inhibitory and excitatory synapses are also detected. On withdrawal day 2 or 14 following 5 days of passive cocaine administration, GABA_A_R-mediated inhibition is decreased at entopeduncular nucleus (EPN)-to-LHb neuronal synapses (measured in brain slices) (Ishikawa and Kenny, 2016; Meye et al., 2016; Figure 1). Furthermore, these EPN-targeted LHb neurons preferentially innervate midbrain (VTA or substantia nigra (SNr)) GABAergic neurons that encode aversion, and normalizing the GABAergic neurotransmission at EPN-to-LHb synapses prevents stress-induced reinstatement to cocaine-associated contexts (Meye et al., 2016).

At excitatory synapses, increases in miniature EPSC (mEPSC) amplitude are observed along with increases in the ratio of currents mediated by AMPA- and NMDA-type glutamate receptors (AMPAR/NMDAR) onto LHb neurons that specifically project to the RMTg (but not the VTA; Maroteaux and Mameli, 2012). This increase in mEPSC amplitude and in AMPAR/NMDAR ratio is likely due to increases in the number of synaptically-expressed AMPARs at this cocaine-withdrawal time point (24 h after two consecutive cocaine intraperitoneal injections; Maroteaux and Mameli, 2012). With cocaine-induced increases in AMPAR levels, the subpopulation of LHb neurons projecting to the RMTg becomes sensitized to synaptic activity and undergoes long-term synaptic potentiation (Maroteaux and Mameli, 2012). These results suggest that specific subpopulations of LHb neurons, with respect to their projection targets, are more sensitive to incoming signals, potentially amplifying select neuronal outputs during cocaine withdrawal.

Increases in LHb neuronal activity are not uniquely correlated with cocaine-induced withdrawal, but are also observed following exposure to ethanol. Withdrawal from ethanol (20% ethanol in a water bottle given intermittently 24 times over 8 weeks) increases spontaneous action potential firing and membrane excitability in LHb neurons measured using *ex vivo* brain slices (Li et al., 2016, 2017). At the synaptic level, the AMPAR/NMDAR ratio and the frequency of sEPSCs are increased during ethanol withdrawal (Li et al., 2016, 2017). Additionally, in an ethanol-induced conditioned taste aversion (CTA) paradigm, the basal firing in LHb neurons is increased *in vivo* when compared to neuronal firing pre-CTA (Tandon et al., 2017).

These results suggest that the increased LHb activity may serve as a neural mechanism to facilitate drug-induced negative affect. Therefore, inhibiting LHb neuronal output could potentially prevent drug-induced anhedonia. To this end, low dose (0.25 g/kg) ethanol-induced conditioned place aversion is reversed to conditioned place preference (CPP) following lidocaine-induced LHb inhibition (Zuo et al., 2017a). In addition, ethanol-induced CTA is attenuated in animals with LHb lesions (Haack et al., 2014; Tandon et al., 2017). Furthermore, cocaine-induced avoidance behaviors in a runway operant paradigm are abolished in animals with lesioned LHb efferents (Jhou et al., 2013).

In summary, withdrawal from drugs of abuse facilitates a correlative response between the intrinsic and synaptic properties of LHb neurons, in that increases in action potential firing and membrane excitability are associated with enhanced glutamatergic transmission and a reduced GABAergic influence. Based on the responses of LHb neurons during localized drug application and during withdrawal, LHb neurons are responsive to drugs of abuse. How these drugs of abuse affect reward learning via the LHb becomes a potentially critical topic to explore.

## How Do Drugs of Abuse Alter LHb-Regulated Reward Prediction or Reward Learning?

The LHb is necessary for updating drug-induced learned behaviors as well as discriminating between behaviors that elicit reward. For example, lesions in the LHb prior to extinction training prevent extinction learning in animals trained to self-administer cocaine (Friedman et al., 2010). Additionally, LHb lesions block an animal’s ability to discriminate between rewarding and non-rewarding predictive cues. This was shown in animals that learned to press an active lever during cocaine availability (signaled by a light cue; Go) and to abstain from lever pressing when cocaine was not available (signaled by the absence of a light cue; No Go; Zapata et al., 2017). Animals with inhibited LHb function showed an inability to discriminate between the predictive cues and continued to press the lever during the No Go cue despite the absence of available reward (Zapata et al., 2017). Furthermore, pharmacologically inhibiting LHb function had no effect on the motivation to seek cocaine, which, in addition to the impaired predictive-cue discrimination mentioned above, led the authors to interpret that the LHb may not only mediate reward updating, but also facilitate suppression of impulsive behaviors (Zapata et al., 2017).

Clinical studies show that patients suffering from cocaine-use disorder have impaired reward learning, compared to healthy controls (Parvaz et al., 2015; Ersche et al., 2016), which can be mediated by changes in the neurocircuits underlying reward prediction (Wrase et al., 2007; Sjoerds et al., 2013; Rose et al., 2014; Deserno et al., 2015). These reward-learning deficits are also observed in preclinical models of addiction which show that reward learning is impaired by drugs of abuse following non-contingent drug administration (Groman et al., 2018) and, as discussed below, contingent drug administration.

**Figure 2:**
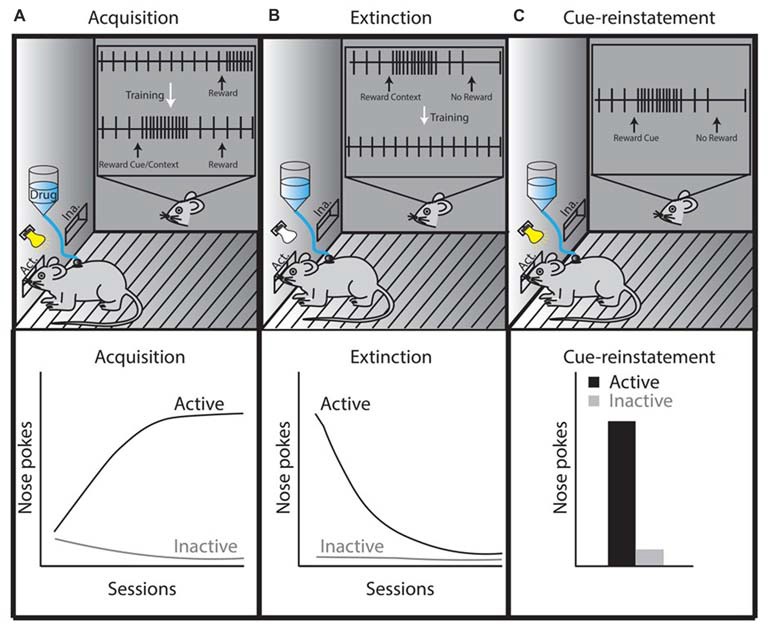
Theoretical illustration of dopamine neuron activity during three different self-administration phases, which is based on phasic dopamine release that occurs in the NAc during drug self-administration, extinction, or reinstatement (Phillips et al., 2003; Stuber et al., 2005). **(A)** In the acquisition or maintenance phases, animals are placed in a specific context in which they learn that active lever presses or nose pokes elicit intravenous drug administration (this is sometimes paired with a cue, which becomes associated with the rewarding properties of the drug). Once acquired, as shown by steady increases in daily active lever or nose pokes (lower left graph, black line), the maintenance phase is initiated and the animals’ active lever presses or nose pokes reach a plateau. The inset in panel **(A)** shows the activity of a midbrain dopamine neuron during drug self-administration. The firing of the dopamine neuron is precisely timed with reward prediction errors (RPEs) such that an unexpected delivery of drug elicits phasic dopamine neuron firing. When these rewards are repeatedly preceded by a cue, phasic dopamine neuron firing shifts from occurring at the time of reward to occurring at the time of exposure to the reward-predicting cue. **(B)** The extinction phase refers to extinguishing the behavior learned during the acquisition/maintenance phase (lower middle graph, black line), which occurs by eliminating drug delivery after the animal completes a learned behavioral response to obtain the drug (e.g., an active lever press or nose poke). This behavior is performed in the absence of a cue in tests requiring cue-reinstatement. The inset in panel **(B)** shows that omitting the expected reward (negative RPE) inhibits dopamine neuron firing. However, drug-associated contexts (i.e., house light, lever or nose poke hole) are likely to induce phasic firing of dopamine neurons until these learned associations are updated during extinction training (McFarland and Ettenberg, 1997; Crombag and Shaham, 2002; Stuber et al., 2005). **(C)** Drug-associated cues reinstate the learned behavior of active nose pokes (lower right graph, panel **C**). If the reward is omitted following the cue presentation (negative RPE), the tonic firing of dopamine-neurons would be inhibited at the time the reward is expected (inset, panel **C**). If this reward omission is repeated, dopamine neurons no longer show phasic firing patterns during reward-predicting cue re-exposure (Schultz et al., 1997; Schultz, 2016). The precise timing of dopamine-neuron firing controls cue-reward learning making the phasic firing of dopamine neurons an important neural mechanism responsible for the adaptability of reward-seeking behaviors.

In the extinction phase of drug self-administration, the drug reward is withheld after performing the learned behavioral response (a negative RPE), likely invoking negative RPE signaling in the LHb and subsequent LHb-induced dopamine neuron inhibition (Figure 2). Therefore, if exposure to drugs of abuse alters LHb function, negative RPE signaling in the LHb would be impaired, thus affecting extinction learning. Animals trained to self-administer drugs of abuse, including psychostimulants, opioids and ethanol, are able to extinguish this learned response (Grimm and See, 2000; Shalev et al., 2001; Richards et al., 2008). This conclusion is not drawn from direct statistical measures, but rather on the clear separation of data points observed between the acquisition phase and the extinction phase of drug self-administration. However, the ability to extinguish a learned response does not imply that extinction learning is fully intact. There are clear time-dependent changes in extinction behavior following withdrawal from heroin and cocaine (this effect is also seen following extinction of sucrose self-administration; Shalev et al., 2001; Grimm et al., 2002), which may rely on changes in neuronal activity of LHb afferents targeting the VTA (Mahler and Aston-Jones, 2012). Future experiments that directly link LHb function and drug-induced extinction learning are still needed in order to demonstrate that the LHb is altered following drug exposure, and that these drug-induced modifications in the LHb are causally associated with reward-learning impairments. Doing so may provide direct evidence that drug-induced modifications in the LHb impair extinction learning and promote relapse to drugs of abuse.

Given that positive RPE is associated with the phasic firing of dopamine neurons, and that negative RPE is associated with LHb signaling, one would expect that the acquisition or maintenance phase of drug self-administration (a positive RPE paradigm) might be independent of LHb control. In agreement with this, it has been shown that LHb lesions do not alter heroin or cocaine self-administration administration during the acquisition or maintenance phases, as LHb lesioned animals seek and obtain similar amounts of drug compared to non-lesioned animals (Wang et al., 2009; Friedman et al., 2010).

## Concluding Remarks

The LHb regulates dopamine neuron firing during negative RPE signaling and provides instructive signals for reward-seeking behaviors (Matsumoto and Hikosaka, 2007; Friedman et al., 2010; Bromberg-Martin and Hikosaka, 2011; Zapata et al., 2017). It is hypothesized that drugs of abuse alter positive RPE signals through drug-induced modifications in midbrain dopamine neurons (Redish, 2004; Keiflin and Janak, 2015). In parallel, drugs of abuse may impact negative RPE signals through drug-induced adaptations in the LHb. Evidence suggests that patients suffering from cocaine-use disorder have impaired negative RPE signaling (Parvaz et al., 2015), which is potentially due to the drug-induced modifications in LHb function (based on preclinical findings; Lecca et al., 2014). These drug-induced modifications include changes in membrane excitability, increases or decreases in excitatory transmission, and increases or decreases in inhibitory transmission, which have lasting impacts on LHb neuronal functions during drug abstinence. Since the LHb is important for encoding negative RPE, and drugs of abuse alter LHb functions, it is possible that negative RPEs are compromised in drug-exposed animals. Although there is some circumstantial evidence that drugs of abuse may affect extinction learning in preclinical models of addiction, direct causal associations between drug-induced LHb modifications and LHb-regulated negative RPE signaling are still lacking.

In addition to regulating negative RPE, the LHb is critically involved in learning and memory processes that are associated with avoidance behaviors (Stamatakis and Stuber, 2012), which are also disrupted in patients suffering from cocaine-use disorder (Ersche et al., 2016). The neuronal mechanisms underlying the impairment of avoidance behaviors are unknown. Preclinical models have shown that psychostimulants facilitate axonal degeneration of the fasciculus retroflexus (a collection of efferent LHb fibers; Carlson et al., 2001; Ellison, 2002; Ciani et al., 2005; Lax et al., 2013), limiting the LHb’s regulation over downstream targets. However, this axonal degeneration has only been detected in animals exposed to psychostimulants; it is unclear whether axonal degeneration of the fasciculus retroflexus is common among other abused substances. Additionally, these avoidance behaviors may be linked to the LHb’s regulation of anxiety disorders through its efferent connections to serotonin neurons in the dorsal raphe nucleus, another brain region signaling reward-context information with implications in addiction (Nakamura, 2013; Müller and Homberg, 2015).

In conclusion, we reviewed evidence supporting the importance of the LHb in regulating reward learning and updating of reward-related information. In preclinical experiments, lesioning the LHb or inhibiting LHb function elicits similar impairments in reward prediction when compared to human patients suffering from drug abuse. However, more work is needed in animal models of drug abuse to understand whether and how drugs of abuse facilitate reward-related learning impairments mediated by LHb function.

## Author Contributions

NG, PN and YD developed the focus of this review. NG and PN wrote the manuscript.

## Conflict of Interest Statement

The authors declare that the research was conducted in the absence of any commercial or financial relationships that could be construed as a potential conflict of interest.
